# Long noncoding RNA LIPH-4 promotes esophageal squamous cell carcinoma progression by regulating the miR-216b/IGF2BP2 axis

**DOI:** 10.1186/s40364-022-00408-x

**Published:** 2022-08-16

**Authors:** Yuhang Xiao, Jinming Tang, Desong Yang, Baihua Zhang, Jie Wu, Zhining Wu, Qianjin Liao, Hui Wang, Wenxiang Wang, Min Su

**Affiliations:** 1 Hunan Clinical Medical Research Center of Accurate Diagnosis and Treatment for Esophageal Carcinoma, Hunan Cancer Hospital and The Affiliated Cancer Hospital of Xiangya School of Medicine, Central South University, Changsha, China; 2 Department of Pharmacy, Xiangya Hospital of Xiangya School of Medicine, Central South University, Changsha, China; 3 Thoracic Surgery Department 2, Hunan Cancer Hospital and The Affiliated Cancer Hospital of Xiangya School of Medicine, Central South University, Changsha, China; 4 Hunan Key Laboratory of Cancer Metabolism, Hunan Cancer Hospital and the Affiliated Cancer Hospital of Xiangya School of Medicine, Central South University, Changsha, China; 5 Key Laboratory of Translational Radiation Oncology, Department of Radiation Oncology, Hunan Cancer Hospital and The Affiliated Cancer Hospital of Xiangya School of Medicine, Central South University, Changsha, China

**Keywords:** Esophageal squamous cell carcinoma, Long noncoding RNA, LIPH-4, miR-216b, IGF2BP2

## Abstract

**Introduction:**

Esophageal squamous cell carcinoma (ESCC) represents a major malignancy with poor clinical outcomes. Long noncoding RNAs (lncRNAs) are known to regulate the development and progression of multiple cancers. However, how lncRNAs are involved in ESCC is currently undefined.

**Methods:**

LIPH-4 levels in ESCC tissue specimens and cells were assessed by qRT-PCR. The biological function of LIPH-4 was examined in cell and animal studies, applying CCK-8, EdU, colony formation and flow cytometry assays as well as xenograft model experiments. The underlying mechanisms of action of LIPH-4 were explored through bioinformatics, luciferase reporter assay, RNA-immunoprecipitation assay and immunoblot.

**Results:**

We identified a novel lncRNA, LIPH-4, which showed elevated amounts in ESCC tissues and positive correlations with increased tumor size and poor prognosis in ESCC patients. Functional studies showed that LIPH-4 promoted the growth, mediated cell cycle progression and inhibited apoptosis in ESCC cells *in vitro*, and promoted tumor growth in mice. In terms of mechanism, LIPH-4 could bind to miR-216b and act as a competing endogenous RNA (ceRNA) to induce the expression of miR-216’s target gene IGF2BP2. LIPH-4 played an oncogenic role in ESCC through the miR-216b/IGF2BP2 axis.

**Conclusions:**

This study suggested that LIPH-4 functions as a novel oncogenic lncRNA by acting as a ceRNA for miR-216b to regulate IGF2BP2, indicating LIPH-4 likely constitutes a prognostic biomarker and therapeutic target in ESCC.

**Supplementary Information:**

The online version contains supplementary material available at 10.1186/s40364-022-00408-x.

## Background

Esophageal cancer is the seventh most commonly diagnosed cancer (604,100 new cases) and the sixth commonest cause of cancer-related mortality (544,076 deaths) around the world in 2020 [1]. Two main EC types are known, including esophageal adenocarcinoma and esophageal squamous cell carcinoma (ESCC), which have quite distinct epidemiology and etiology [2]. ESCC, which usually originates from the lining of the esophageal squamous epithelium and occurs predominantly in the upper and mid-esophagus, is the predominant form of EC worldwide. In China, EC is the fourth leading cause of cancer-related death, more than 90% of the EC cases are ESCCs [3]. Despite the remarkable improvement in treatment, ESCC prognosis remains poor, with 5-year survival below 20%, mostly because of late diagnosis, common metastasis and rapid tumor progression [4, 5]. Additionally, the precise genetic and molecular mechanisms of ESCC remain unknown [6]. Consequently, a better understanding of the mechanism underlying ESCC formation and progression is required for improving early diagnosis and therapy.

Long non-coding RNAs (lncRNAs) represent RNAs with > 200 nucleotides lacking overt protein-coding capacity [7]. LncRNAs exert their functions via diverse mechanisms, e.g., recruiting chromatin modification complexes to the chromatin, interacting with RNA including microRNAs (miRNAs), and interacting with proteins [8]. Mounting evidence suggests lncRNAs might be critical modulators in multiple biological processes, including development, differentiation and carcinogenesis [9]. For instance, lncRNA CASC9 has high expression in ESCC and induces metastasis by increasing LAMC2 amounts via interaction with the CREB-binding protein [10]. LncRNA APCDD1L-AS1 is identified as the most significantly upregulated lncRNA in icotinib-resistant lung adenocarcinoma cells, and promotes icotinib resistance through inhibiting autophagic degradation of EGFR via the miR-1322/miR-1972/miR-324-3p-SIRT5 axis [11]. In addition, most recent evidences have shown some lncRNAs contain short open reading frames that can be translated into biologically active small peptides [12]. The lncRNA HOXB-AS3 binds ribosomes and encodes a highly conserved 53-aa peptide named HOXB-AS3, which can act as a tumor suppressor and inhibit colon cancer cell proliferation, migration, and invasion [13]. A novel small peptide SPAR, which encoded by the lncRNA LINC00961, is localized to the late endosome/lysosome and interacts with the lysosomal v-ATPase to negatively regulate mTORC1 activation [14]. Although multiple lncRNAs contribute to tumor formation and progression in ESCC, the involvement of most lncRNAs in ESCC remains undefined.

In our previous report, we performed lncRNA microarray assays to analyze lncRNAs with deregulated expression in ESCC tissues in comparison to adjacent noncancerous tissues, and identified some specific lncRNAs that might participate in ESCC formation and progression [15]. A new highly expressed lncRNA, LIPH-4 (NONHSAT093780), was shown to rank the top 10 upregulated lncRNAs. Herein, lncRNA LIPH-4 was identified in ESCC and its levels, clinical importance, function and underpinning mechanism in ESCC were examined.

## Materials and methods

### Clinical samples

Totally 53 clinical ESCC and adjacent noncancerous tissue samples (> 2.0 cm from the tumor edge) were obtained during surgery at the Hunan Cancer Hospital of Central South University from January 2015 and December 2016. No patients underwent preoperative therapy. The samples underwent snap freezing in liquid nitrogen and stored at -80 °C. The collection and utilization of tissue samples had approval from the ethics committee of Hunan Cancer Hospital, conforming with current regulations. Each participant provided signed informed consent. The clinicopathologic features of all patients with ESCC are listed in Table 1.

**Table 1:** Relationship between LIPH-4 expression and clinicopathologic parameters of 53 ESCC patients

Characteristics	Number of case	lnc-LIPH-4		*P* value
		High(*n* = 26)	Low(*n* = 27)	
Age(years)				0.8967
< 60	28	13	15	
≥ 60	25	13	12	
Tumor size				0.0030**
< 5 cm	34	11	23	
≥ 5 cm	19	15	4	
Histological grade				0.6963
low and Middle	20	11	9	
high	33	15	18	
Tumor invasion depth				0.1763
T1 + T2	18	6	12	
T3 + T4	35	20	15	
Lymph node metastasis				0.3997
negative	23	9	13	
positive	30	17	13	
Clinical stage				0.8967
I and II	28	14	14	
III and IV	25	12	13	

### Cell lines

Human ESCC cells (TE-1, Eca109, KYSE30, KYSE150, KYSE410 and KYSE510), and human normal esophageal epithelial HET-1A cells were obtained from the Institute of Cell Research, Chinese Academy of Sciences (Shanghai, China). KYSE30 cells were cultured in Dulbecco’s modified Eagle’s medium (DMEM) with 10% fetal bovine serum (FBS, Hyclone, USA) and 1% PenStrep (100 U/mL Penicilium and 100 μg/mL Streptomycin), the other cell lines were cultured in RPMI 1640 containing 10% FBS and 1% PenStrep. All cells were maintained in a humid atmosphere containing 5% CO2 at 37 °C.

### Lentivirus construction and cell transfection

For stable overexpression of LIPH-4 and Insulin-like growth factor 2 mRNA-binding protein 2 (IGF2BP2), LIPH-4 and IGF2BP2 expression plasmids were constructed by inserting the related cDNA sequences into the Ubi-MCS-SV40-EGFP-IRES-puro lentiviral vector (Genechem, China). For stable knockdown of LIPH-4 and IGF2BP2, siRNA constructs for LIPH-4 or IGF2BP2 were obtained by cloning the DNA sequences targeting LIPH-4 or IGF2BP2 into the hU6-MCS-CBh-gcGFP-IRES-puromycin plasmid, which were introduced in a lentiviral vector (Genechem, China). For lentivirus transfection, cells were incubated with viral particles overnight with 10 μg/mL polybrene (Sigma-Aldrich, USA). Then, the specimens were incubated for 14 days with 2 μg/ml puromycin to establish stable transfectants. MiR-216b-5p mimics and inhibitors were provided by Hanbio (Shanghai, China) and transient transfection utilized lipofectamine 3000 (Invitrogen, USA) as directed by the manufacturer. The sequences of siRNA were listed in Supplementary Table 1.

### Cell counting Kit-8 (CCK-8) assay

ESCC cells underwent seeding in a 96-well plate at 2 × 10^3^/well and incubated for 4 days. Daily, 10 μl of CCK-8 solution was supplemented per well and incubated for 1 h at 37 °C before absorbance measurement at 450 nm.

### Colony formation assay

Totally 10^3^ ESCC cells underwent seeding into a 35-mm dish and incubation for 14 days. Cell colonies were fixed with 4% formalin for 15 min and stained with 0.1% crystal violet. Colonies with > 50 cells were imaged by light microscopy and counted with Image J.

### 5-ethynyl-2’-deoxyuridine (EdU) assay

The EdU assay was carried out with EdU Cell Proliferation Kit with Alexa Fluor 555 (Epizyme, China) as directed by the manufacturer. Briefly, ESCC cells were cultured in a 24-well plate overnight. Then, the EdU reagent was added for 2 h at 37 °C, followed by cell fixation (4% formalin), permeabilization (0.5% Triton-X-100, Sigma) and Hoechst 33,342 counterstaining. A fluorescence microscope was utilized for analysis.

### Flow cytometry assay

Cells underwent culture in 6-well plates for 24 h culture, followed by overnight fixation at 4 °C in 70% ethyl alcohol. The specimens were assessed with Cell Cycle and Apoptosis Analysis Kit (Beyotime, China) as directed by the manufacturer. Cell cycle analysis was carried out with a FACS Calibur flow cytometer (BD Biosciences, USA).

Cell culture was performed in 6-well plates for 24 h, and the apoptotic rate was examined with the Annexin-VFITC apoptosis detection kit (BD, USA) according to the inserted protocol. Analysis was performed with a FACS Calibur flow cytometer.

### RNA extraction and quantitative reverse transcription polymerase chain reaction (qRT-PCR)

Total RNA extraction from tissue and cell samples was carried out with TRIzol (Invitrogen, USA) following the kit’s protocol. LncRNAs and mRNAs were assessed with a SYBR Green PCR Kit (Takara, Japan), using GAPDH as an internal control gene. A miDETECT A Track Kit (RiboBio, China) was utilized to detect microRNAs, with U6 for normalization. The 2^−△△Ct^ method was utilized for data analysis. Primer sequences were listed in Supplementary Table 2.

### Subcellular fractionation

Cytoplasmic and nuclear fractions were obtained with a PARIS Kit (Invitrogen, USA) as directed by the manufacturer, and qRT-PCR was performed to quantitate LIPH-4 mRNA, with U6 and GAPDH as nuclear and cytoplasmic controls, respectively.

### Western blot

Total protein was obtained from cell samples with the RIPA buffer (Beyotime, China) supplemented with protease inhibitor cocktail tablet (Roche, Switzerland). Western blot was carried out with rabbit antibodies targeting IGF2BP2 (1:5000, Abcam, UK), cyclinD1 (1:1000, Abways, china), AKT (1:1000, Abways, china), p-AKT (1:1000, Abways, china), and GAPDH (1:5000, ZENbio, China) antibodies. HRP-conjugated IgG (1:5000, Beyotime, China) was utilized as secondary antibodies. The ECL-Plus reagent (Millipore, USA) was utilized for development. GAPDH was used for normalization.

### Xenograft model

4–5-week-old female BALB/c nude mice (*n* = 6/group) were housed under specific pathogen-free conditions. To establish the xenograft model, stably transfected cells (5 × 10^6^ in 0.2 ml PBS containing 10% Matrigel (BD Biosciences, USA) were injected by the subcutaneous route into the mouse armpit. Each tumor was measured every 3 days with digital calipers to derive tumor volume as length × width2/2. Euthanasia was performed at 4 weeks, and the final volume and weight of each tumor were assessed. The tumors were paraffin embedded, followed by hematoxylin and eosin (H&E) staining or immunohistochemistry (IHC). Experiments involving animals had approval from the Animal Ethics Committee of Hunan cancer hospital, following The Guidelines for the Care and Use of Laboratory Animals.

### Luciferase reporter assay

Bioinformatics tools were utilized for predicting potential miR-216b binding sites of LIPH-4- and IGF2BP2-3ʹ-UTR (Starbase v2.0, RegRNA2.0, miRcode and microRNA.org). Human 293 T cells were co-transfected with 160 ng of empty pmirGLO-LIPH-4-wt/mut or pmirGLO-IGF2BP2-wt/mut and 5 pmol miR-216b mimic or miR-NC utilizing Lipofectamine 3000 (Invitrogen, USA) strictly following the manufacturer’s recommendations. After 48 h of incubation, Dual-Luciferase Reporter Assay System (Promega, USA) was utilized for luciferase activity assessment in triplicates experiments.

### RNA immunoprecipitation (RIP) assay

An EZ Magna RNA immunoprecipitation Kit (Millipore, USA) was applied as directed by the manufacturer. In brief, KYSE510 cells underwent lysis with RIP lysis buffer. The resulting cell lysates were added to magnetic beads linked to anti-Ago2 antibodies (Millipore, USA) or control anti-IgG for overnight incubation at 4 °C. The immunoprecipitated RNA was isolated and assessed by qRT-PCR.

### Statistical analysis

Data are mean ± standard deviation from three assays or more, performed independently. SPSS 18.0 (SPSS, USA) and GraphPad Prism 6 (GraphPad, USA) were utilized for data analysis. The Pearson chi-square test was utilized to assess associations of LIPH-4 expression with clinicopathological variates. Kaplan–Meier curve analysis was used to assess survival, and the log-rank test was used for comparisons. Differences between groups were analyzed by Student’ s t-test. *P* < 0.05 was deemed statistically significant.

## Results

### LIPH-4 upregulation in ESCC patients correlates with poor survival

**Fig. 1:**
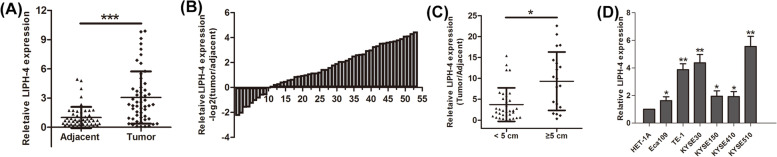
Expression of LIPH-4 in ESCC tissues, cells and its clinical significance. **A**-**B** Relative expression levels of LIPH-4 in ESCC and adjacent noncancerous tissue samples assessed by qRT-PCR (*n* = 53). **C** The correlation between LIPH-4 expression and tumor size. (D) Relative expression levels of LIPH-4 in human ESCC and esophageal epithelial HET-1A cells (*n* = 3). *P < 0.05, **P < 0.01, ***P < 0.001

We previously carried out RNA transcriptome sequencing of five ESCC and adjacent noncancerous tissue specimens by microarray experiments. Totally 1,032 upregulated lncRNAs (> twofold, *p* < 0.05) and 1,344 downregulated lncRNAs were identified(< 0.5-fold, *p* < 0.05). Among these lncRNAs, LIPH-4, which is a 472-bp transcript localized in human chromosome 3q27.2, ranked as one of the top remarkably upregulated lncRNAs. To further examine LIPH-4 expression, qRT-PCR was performed to assess LIPH-4 amounts in 53 paired ESCC tissue and adjacent noncancerous tissue specimens. LIPH-4 was significantly overexpressed in 81.13% (43 of 53 paired) of the ESCC tissue samples versus the respective normal tissue samples (*P* < 0.001, Fig. 1A and B). Then, the associations of LIPH-4 expression with clinicopathological features of ESCC cases were evaluated. We found LIPH-4 expression was associated with tumor size (*P* < 0.05; Fig. 1C). Patients with tumor size exceeding 5 cm had elevated LIPH-4 amounts, whereas those with tumor size below 5 cm had reduced LIPH-4 expression (Table 1). No relationships were found between LIPH-4 expression and other clinicalparameters, including age (< 60, ≥ 60), histological grade (low and middle, high), tumor invasion depth (T1 and T2, T3 and T4), and lymph node metastasis (negative, positive).

To examine the prognostic potential of LIPH-4, overall survival (OS) rates were analyzed. Kaplan–Meier curve analysis demonstrated elevated LIPH-4 amounts were markedly associated with decreased overall survival (*P* < 0.01). Overall, the above data suggested elevated LIPH-4 constituted a factor reflecting tumor progression and reduced survival in ESCC.

### LIPH-4 induces ESCC cell growth in vitro

To further examine LIPH-4 expression, we determined the gene expression of LIPH-4 by qRT-PCR in ESCC cells, including TE-1, Eca109, KYSE30, KYSE150, KYSE410 and KYSE510 cell lines alongside the noncancerous esophageal epithelial HET-1A cells. The results demonstrated LIPH-4 amounts were markedly elevated in all six ESCC cells compared with HET-1A cells (all *p* < 0.05, Fig. 1D). The KYSE510 cell line with the highest LIPH-4 expression levels and the KYSE150 cell line with relatively low LIPH-4 expression levels were selected for subsequent assays.

**Fig. 2:**
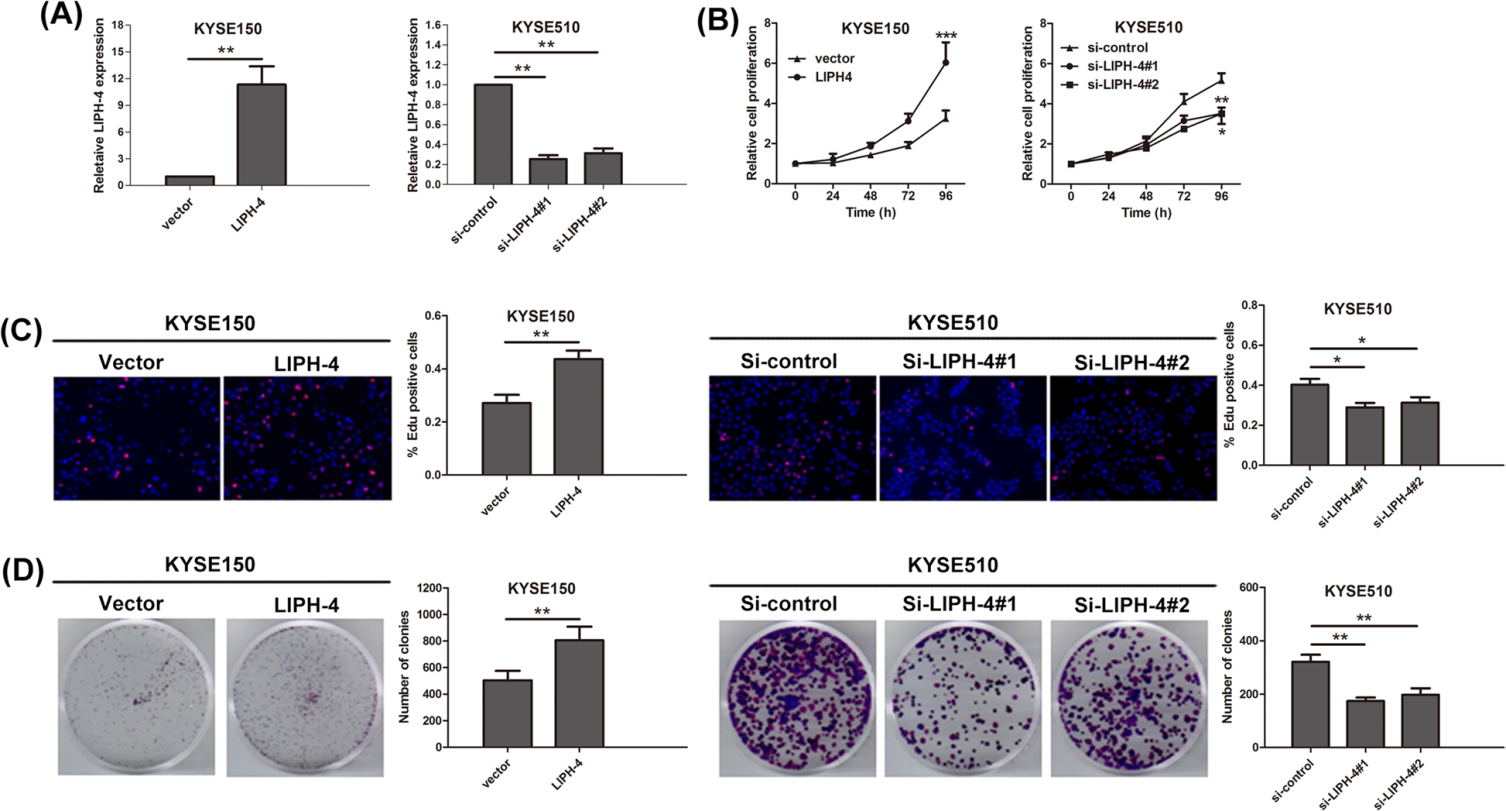
LIPH-4 affects ESCC progression in vitro. **A** LIPH-4 expression was detected by qRT-PCR in KYSE150 cells transfected with LIPH-4, and KYSE510 cells transfected with two LIPH-4 siRNAs (*n* = 3). Cell proliferation was analyzed by **B** CCK-8, **C** immunofluorescence analysis with Edu, and **D** colony formation assay (*n* = 3). *P < 0.05, **P < 0.01, ***P < 0.001

To examine the biological function of LIPH-4 in ESCC progression, loss- and gain-of-function assays were carried out. In KYSE510 and KYSE150 cells, siRNA-induced silencing and plasmid-based overexpression were performed to manipulate LIPH-4 expression, and qRT-PCR was performed for validation (Fig. 2A). Functionally, CCK-8 assay demonstrated LIPH-4 overexpression promoted KYSE150 cell proliferation, whereas LIPH-4 knockdown reduced KYSE510 cell proliferation, in comparison with that of their counterpart controls (Fig. 2B). In agreement, the EdU assay revealed LIPH-4 silencing reduced ESCC cell proliferation, whereas its overexpression promoted ESCC cell proliferation (Fig. 2C). Additionally, the clonogenic assay showed LIPH-4 overexpression increased the clonogenic survival of KYSE150 cells, and LIPH-4 silencing remarkably decreased clone formation in KYSE510 cells (Fig. 2D).

### LIPH-4 affects cell cycle progression and apoptosis in vitro

**Fig. 3:**
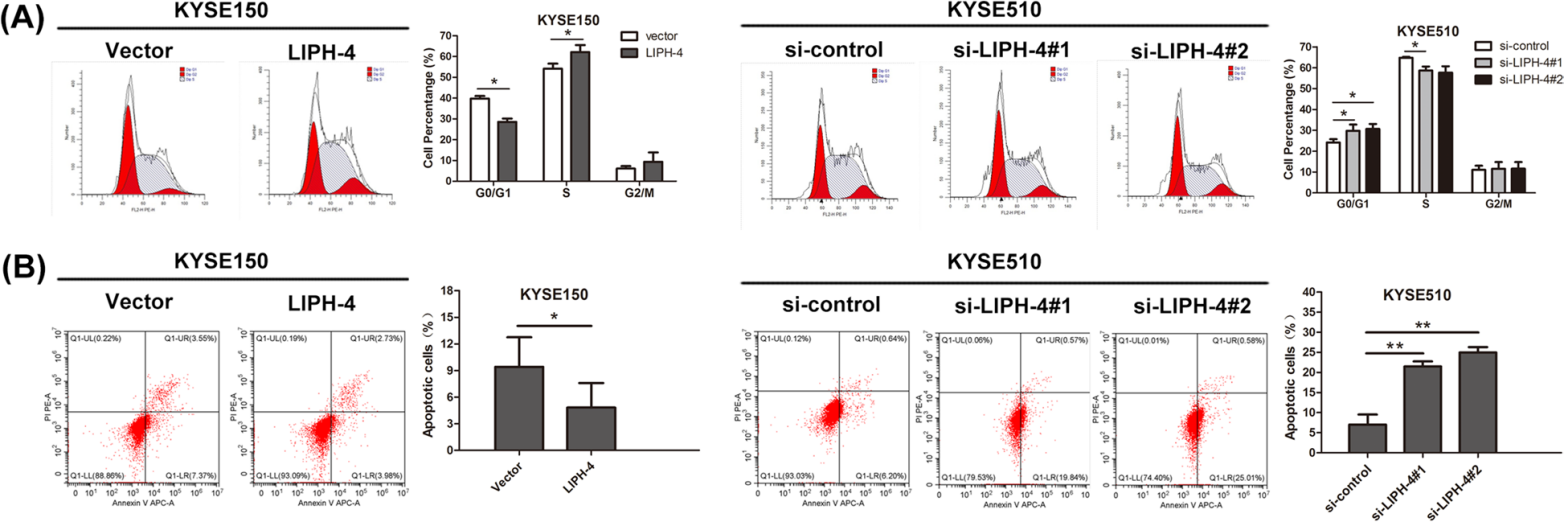
LIPH-4 affects ESCC cell cycle and apoptosis in vitro. **A** Apoptotic rates of ESCC cells after transfection with LIPH-4 or si-LIPH-4, examined by FACS analysis (*n* = 3). **B** The proportion of ESCC cells in G1, S, and G2/M phases after transfection with LIPH-4 or si-LIPH-4, examined by FACS analysis (*n* = 3). *P < 0.05, **P < 0.01, ***P < 0.001

Flow cytometry was then performed to analyze the impact of LIPH-4 on cell cycle progression and apoptosis. The results showed LIPH-4 overexpression in KYSE150 cells induced the G1 to S phase cell cycle transition, whereas LIPH-4 knockdown in KYSE510 cells increased the amount of cells in the G0/G1 phase, in comparison with respective negative controls (Fig. 3A). Meanwhile, the apoptotic rate was markedly decreased after LIPH-4 overexpression in KYSE150 cells, and elevated following LIPH-4 silencing in KYSE510 cells (Fig. 3B).

### LIPH-4 sponges miR-216b

**Fig. 4:**
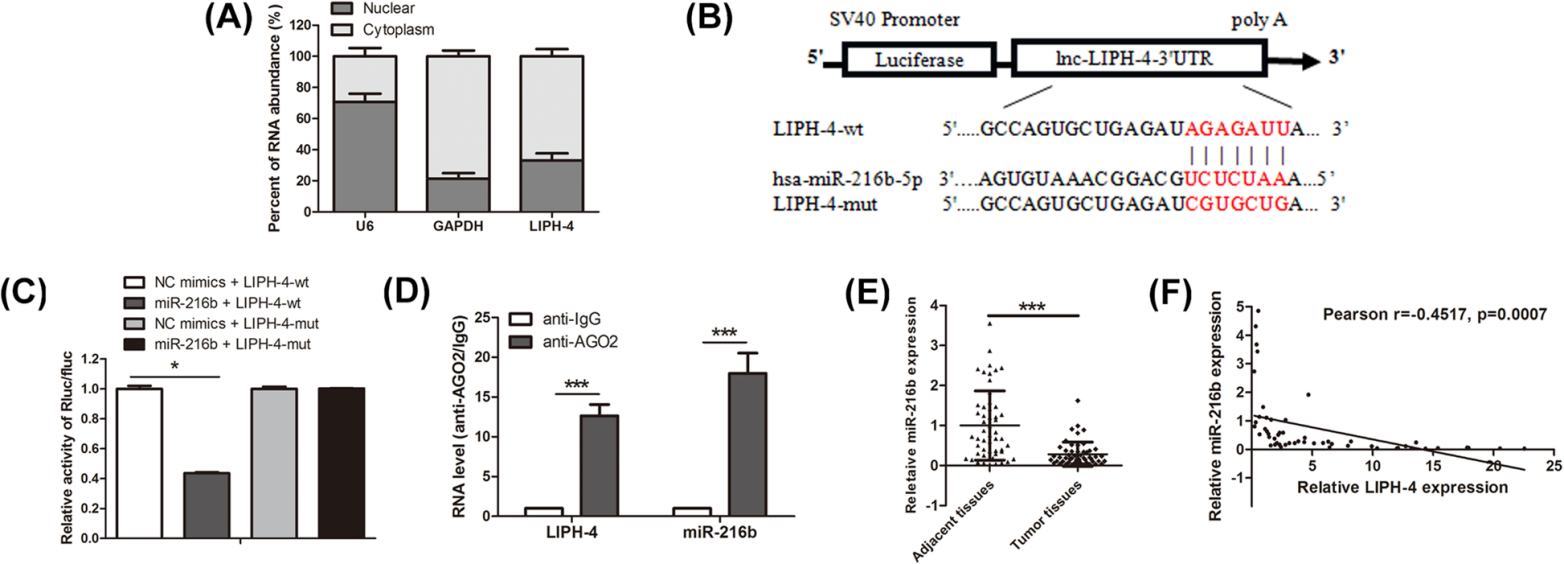
LIPH-4 acts as a sponge of miR-216b in ESCC cells. **A** Relative LIPH-4 expression levels in nuclear and cytosolic fractions of KYSE510 cells, measured by qPCR. U6 (nuclear retained) and GAPDH (exported to cytoplasm) were used as controls (*n* = 3). **B** Sequence alignment of miR-216b with binding sites in the wild-type (LIPH-4-wt) and mutant-type regions of LIPH-4 (LIPH-4-mut). **C** Relative luciferase activity in 293 T cells was assessed after co-transfection with the reporter plasmid (LIPH-4-wt or LIPH-4-mut) and miRNAs (miR-216b or NC mimics) (*n* = 3). **D** RIP assay was performed with an anti-Ago2 antibody. The levels of LIPH-4 and miR-216b were determined by qRT-PCR and presented as fold enrichment in Ago2 relative to IgG immunoprecipitates (*n* = 3). **E** Relative expression levels of miR-216b in ESCC and adjacent noncancerous tissue samples assessed by qRT-PCR (*n* = 53). **F** Negative correlation between miR-216b and LIPH-4. *P < 0.05, **P < 0.01, ***P < 0.001

To explore the mechanism by which LIPH-4 controls ESCC progression, we firstly assessed its localization, because the function of a lncRNA depends on its subcellular distribution [16]. By analyzing the cytoplasmic and nuclear RNA fractions of ESCC cells, LIPH-4 was mainly detected in the cytoplasmic fraction (Fig. 4A). Multiple cytosolic lncRNAs function as miRNA sponges via competitive binding to miRNAs [17]. Thus, bioinformatics software were employed for predicting miRNAs that could potentially target LIPH-4. As a result, miR-216b-5p, an important tumor suppressor [18], was predicted to have putative LIPH-4 binding sites (Fig. 4B). To further validate the binding of miR-216b to LIPH-4, luciferase reporter vectors containing wild-type LIPH-4 (pmirGLO-LIPH-4-wt) and LIPH-4 with mutation of the miR-216b-binding site (pmirGLO-LIPH-4-mut) were constructed. Figure 4C shows 293 T cells co-transfected with the miR-216b mimic and LIPH-4-wt, but not LIPH-4-mut, exhibited significantly decreased luciferase activity. In addition, RIP assay results showed LIPH-4 and miR-216b were significantly enriched in AGO2-containing micro-ribonucleoprotein complexes, suggesting that the AGO2 protein bound directly to LIPH-4 and miR-216b in ESCC cells (Fig. 4D). Moreover, qRT-PCR demonstrated miR-216b expression was reduced in 53 ESCC tissue pairs (*P* < 0.001, Fig. 4E), and Pearson analysis revealed miR-216b and LIPH-4 amounts were negatively correlated (*r* = -0.4517, *p* = 0.0007, Fig. 4F). Taken together, these findings indicated LIPH-4 sponged miR-216b.

### LIPH-4 acts as a ceRNA in IGF2BP2 regulation

**Fig. 5:**
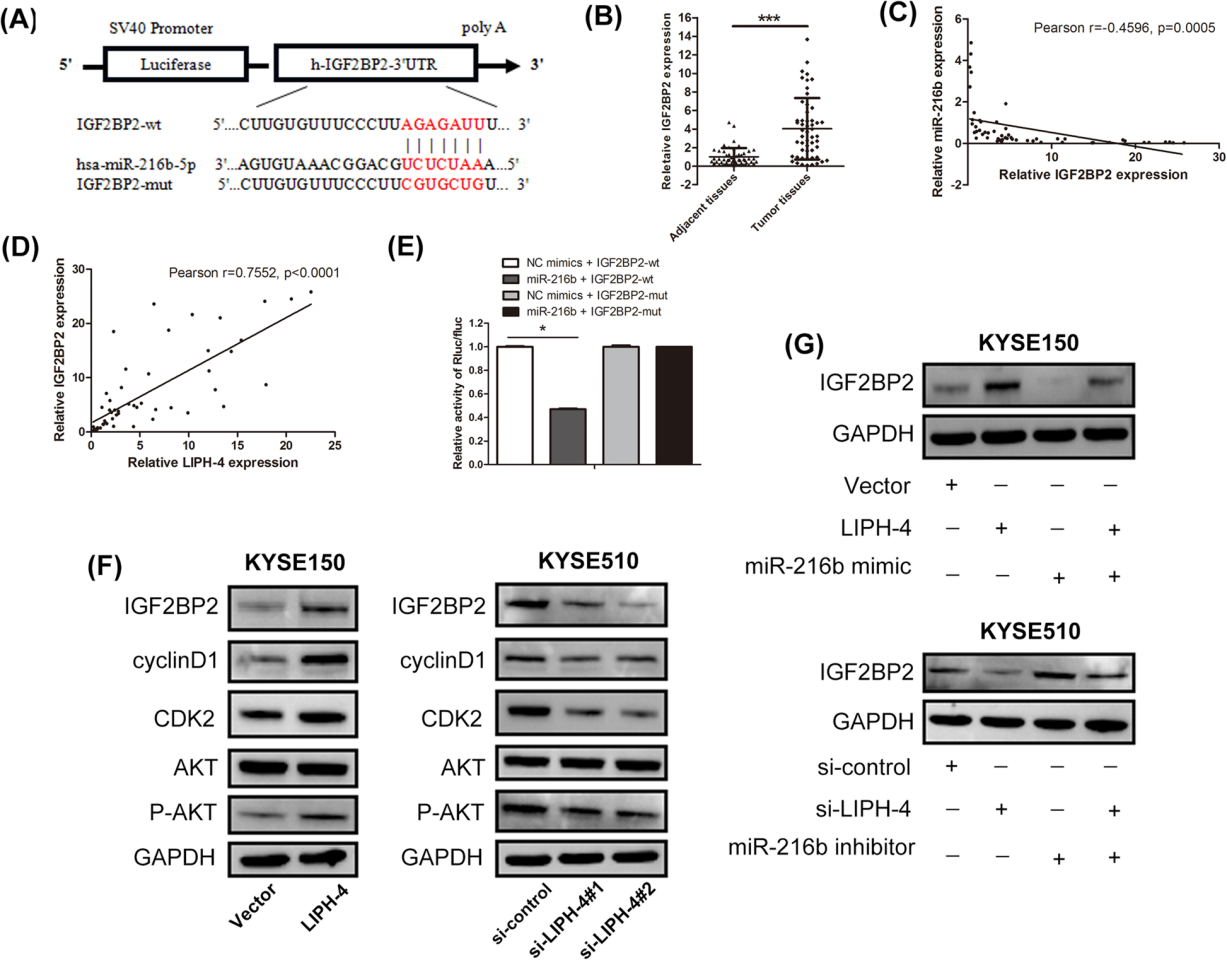
LIPH-4 regulates IGF2BP2 through miR-216b in ESCC cells. **A** Sequence alignment of miR-216b with binding sites in the wild-type (IGF2BP2-wt) and mutant-type regions of IGF2BP2 (IGF2BP2-mut). **B** Relative expression levels of IGF2BP2 in ESCC and adjacent noncancerous tissue samples assessed by qRT-PCR (*n* = 53). **C** Negative correlation between miR-216b and IGF2BP2. **D** Positive correlation between LIPH-4 and IGF2BP2. **E** Relative luciferase activity in 293 T cells was assessed after co-transfection with the reporter plasmid (IGF2BP2-wt or IGF2BP2-mut) and miRNAs (miR-216b or NC mimics) (*n* = 3). **F** IGF2BP2, cyclinD1, AKT, p-AKT protein levels in KYSE150 cells transfected with LIPH-4 and in KYSE510 cells transfected with si-LIPH-4, assessed by immunoblot. **G** IGF2BP2 protein levels in KYSE150 cells transfected with LIPH-4 and miR-216b mimic, and KYSE510 cells transfected with si-LIPH-4 and miR-216b inhibitor, measured by immunoblot. *P < 0.05, **P < 0.01, ***P < 0.001

We further investigated the mechanism by which LIPH-4 mediates the progression of ESCC. LncRNAs participate in the lncRNA-miRNA-mRNA crosstalk, and function by affecting the respective downstream targets [19, 20]. Bioinformatic analysis using public prediction algorithms indicated a possible binding site of miR-216b at the 3’UTR of IGF2BP2 (Fig. 5A). qRT-PCR assays revealed IGF2BP2 level was much higher in ESCC tissues than that in adjacent tissues (Fig. 5B). Pearson analysis showed a negative correlation between miR-216b and IGF2BP2 in ESCC samples (*r* = -0.4596, *p* = 0.0005, Fig. 5C), and LIPH-4 and IGF2BP2 were positively correlated (*r* = 0.7552, *p* < 0.0001) (Fig. 5D). Next, dual luciferase reporter assays showed IGF2BP2 was directly targeted by miR-216b (Fig. 5E). Following that, western blot analysis showed that LIPH-4 overexpression upregulated IGF2BP2 expression, whereas LIPH-4 knockdown downregulated IGF2BP2 expression in ESCC cells. (Fig. 5F). In addition, the IGF2BP2 downstream effector phosphorylated AKT (S473) and the cell cycle-related protein cyclin D1 were increased after LIPH-4 overexpression. The levels of p-AKT (S473), cyclin D1 were diminished following knockdown of LIPH-4.

To confirm that the above mRNA and lncRNA competed for miRNA binding, IGF2BP2 amounts were assessed. We observed decreased IGF2BP2 expression in KYSE150 cells upon transfection with the miR-216b mimic and increased IGF2BP2 amounts in KYSE510 cells after transfection with miR-216b inhibitor in comparison with the respective control groups, as assessed by immunoblot (Fig. 5G). Furthermore, the increased IGF2BP2 protein levels following LIPH-4 overexpression were reversed after co-transfection with the miR-216b mimic. Additionally, upon co-transfection with si-LIPH-4 and the miR-216b inhibitor, si-LIPH-4 induced downregulation of IGF2BP2 was reversed. The above results suggested that LIPH-4 functioned as a ceRNA for regulating IGF2BP2 by sponging miR-216b.

### IGF2BP2 is responsible for the tumor-promoting effects of LIPH-4

**Fig.6:**
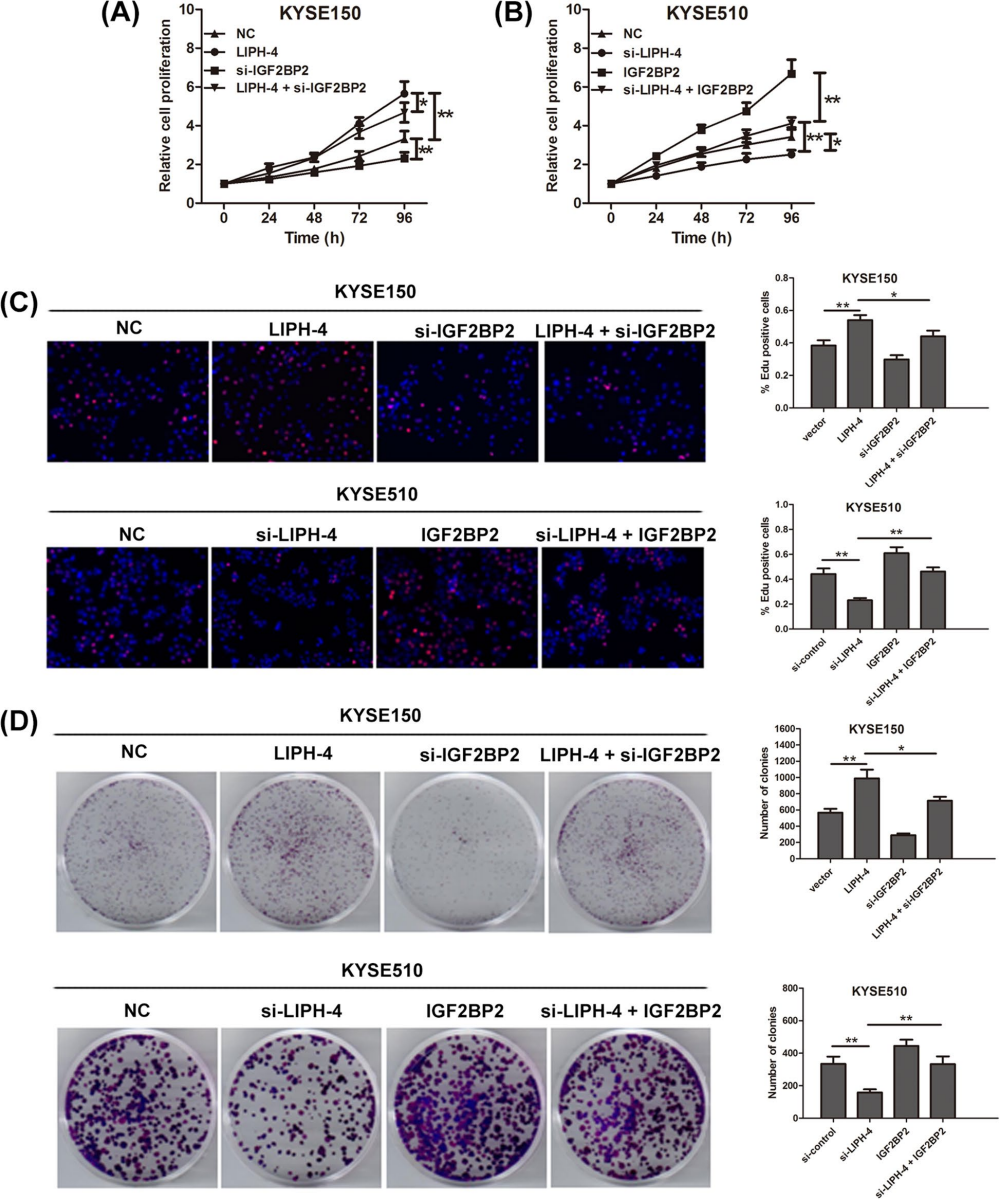
LIPH-4 regulates ESCC progression through IGF2BP2. Cell proliferation was analyzed by **A** CCK-8, **B** immunofluorescence analysis with Edu, and **C** colony formation assay in KYSE150 cells transfected with LIPH-4 and si-IGF2BP2, and in KYSE510 cells transfected with si-LIPH-4 and IGF2BP2 (*n* = 3). *P < 0.05, **P < 0.01, ***P < 0.001

To investigate whether IGF2BP2 contributed to LIPH-4-induced ESCC cell growth, rescue assays were carried out. The CCK-8 and Edu assays demonstrated IGF2BP2 knockdown partially abolished LIPH-4 overexpression-induced growth acceleration in KYSE150 cells (Fig. 6A and B). By contrast, overexpression of IGF2BP2 recovered the proliferative ability of LIPH-4 stable knockdown KYSE510 cells. Similarly, the clone formation assay demonstrated IGF2BP2 silencing partly rescued LIPH-4 overexpression-induced reduction of clonogenic survival, and the effect of LIPH-4 knockdown on the clonogenic survival of KYSE510 cells was also partially rescued by overexpression of IGF2BP2 (Fig. 6C). Jointly, these findings indicated LIPH-4 promoted ESCC progression through regulation of IGF2BP2.

### LIPH-4 promotes growth of ESCC in vivo

**Fig.7:**
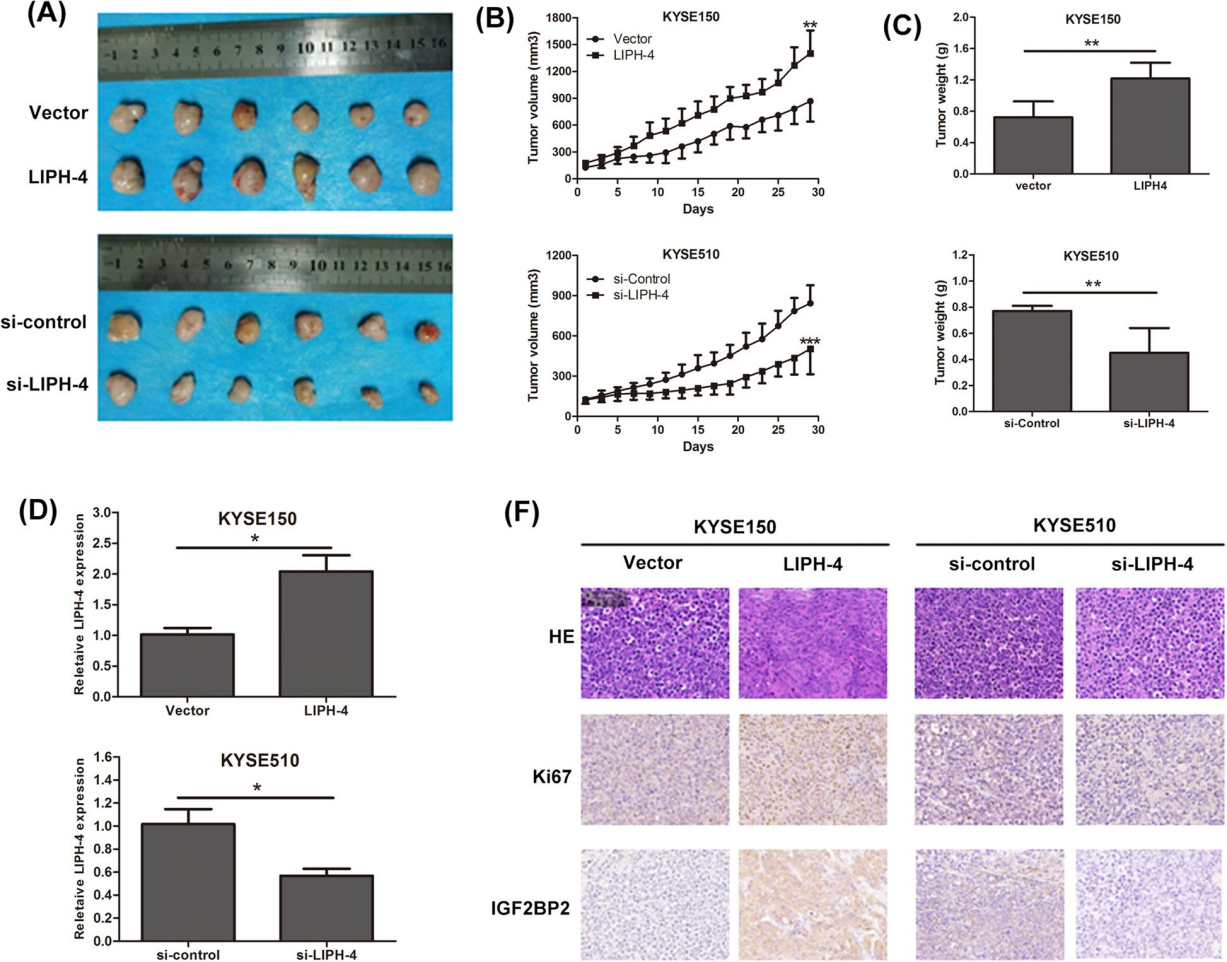
LIPH-4 mediates ESCC progression *in vivo*. **A** Representative images, **B** volumes, **C** weights of tumors formed in nude mice injected with KYSE150 cells transfected with LIPH-4 and KYSE510 cells transfected with si-LIPH-4 (*n* = 6). **D** LIPH-4 expression in xenograft tumor samples detected by qRT-PCR (*n* = 6). (E) Ki-67 and IGF2BP2 expression in xenograft tumors detected by immunohistochemistry (magnification =  × 200, Scale bar = 50 μm). *P < 0.05, **P < 0.01, ***P < 0.001

To further confirm LIPH-4’s oncogenic role in vivo, stably LIPH-4 or si-LIPH-4 transfected ESCC cells were administered by subcutaneous injection into BALB/c nude mice for constructing a xenograft model. Unsurprisingly, LIPH-4 overexpression starkly induced the tumor growth of KYSE150 cells in nude mice, with remarkably increased tumor size and weight in comparison with the negative control group. Conversely, significant reductions of both tumor volume and weight were observed in the LIPH-4 knockdown KYSE510 group in comparison with the control group (Fig. 7A-C). Xenograft tumors generated from LIPH-4-silenced cells had lower LIPH-4 expression and tumors formed from LIPH-4 overexpressing cells had higher LIPH-4 expression than that in tumors from control cells (Fig. 7D). Additionally, IHC demonstrated the xenografts from LIPH-4 knockdown cells had reduced Ki67 and IGF2BP2 expression, whereas the LIPH-4 overexpression group showed elevated Ki67- and IGF2BP2-positivity rates compared with control cells (Fig. 7E). Taken together, the above findings suggested that LIPH-4 significantly promoted the tumor growth of ESCC in vivo.

## Discussion

Increasing evidence reveals lncRNAs play a critical role in ESCC progression [21]. Besides well-characterized lncRNAs, potential critical lncRNAs mediating ESCC formation and progression should be examined. In our previous report, lncRNA microarray assay was carried out for analyzing the profiles of ESCC tissues in comparison with adjacent noncancerous tissues and identified a novel upregulated lncRNA, LIPH-4 [15]. In the present work, the function and mechanism of LIPH-4 in ESCC were examined. We demonstrated that LIPH-4 amounts were increased in ESCC tissue specimens and cells. Enhanced LIPH-4 expression showed positive correlation with larger tumor size and reduced OS in ESCC. Therefore, this work identified a novel ESCC-associated lncRNA LIPH-4, which was positively associated with reduced patient survival in ESCC.

Loss- and gain-of-function experiments suggested LIPH-4 silencing suppressed cell proliferation, colony formation and cell cycle progression, while inducing apoptosis in cultured ESCC cells. Meanwhile, LIPH-4 overexpression produced the opposite effects. In vivo xenograft assays demonstrated LIPH-4 knockdown reduced ESCC tumor growth in mice, while LIPH-4 overexpression promoted ESCC tumor growth. Jointly, the above findings indicate an oncogenic role for LIPH-4 in ESCC.

In terms of mechanism, emerging evidence demonstrates that the modulatory effects of lncRNAs strongly depend upon their location in cells [22]. lncRNAs that located in cytoplasm can serve as a natural miRNA sponge, subsequently regulating miRNA targets and modulating their functions [23, 24]. We found that LIPH-4 is primarily expressed in the cytoplasm of ESCC cells. Next, bioinformatics analysis revealed that miR-216b might have potential LIPH-4 binding sites. MiR-216b is considered a tumor suppressor in diverse malignancies [25, 26]. In agreement, LIPH-4 and miR-216b levels were inversely correlated in ESCC. Furthermore, luciferase reporter and RIP assays confirmed miR-216b as a direct LIPH-4 target.

IGF2BP2, belonging to the conserved IGF2 mRNA binding protein family, regulates subcellular RNA localization, stability and translation [27, 28]. It is known IGF2BP2 is highly expressed and induces progression in multiple malignancies [29], including esophageal cancer [30, 31]. Here, IGF2BP2 was shown to directly target miR-216b in ESCC cells. IGF2BP2 was positively and negatively correlated with LIPH-4 and miR-216b in ESCC tissue samples, respectively. Notably, LIPH-4 upregulated IGF2BP2 in ESCC cells, while miR-216b had the opposite effect. Importantly, the effect of ESCC cell proliferation induced by LIPH-4 overexpression or knockdown were reversed by IGF2BP2 silencing or restoration. Overall, this study provides a new evidence that the LIPH-4/miR-216b/IGF2BP2 axis is involved in ESCC growth.

## Conclusions

In summary, our findings identified a novel upregulated lncRNA LIPH-4, which functions as an oncogenic lncRNA during ESCC progression and reveals a ceRNA regulatory pathway in which LIPH-4 upregulates IGF2BP2 expression by sponging miR-216b. Collectively, these data suggested that LIPH-4 might be a potential prognostic biomarker and therapeutic target for ESCC.

## Supplementary Information

**Additional file 1: Supplementary Table 1.** Primer sequences for qRT-PCR. **Supplementary Table 2.** Sequences of siRNA.

## Data Availability

Data related to this paper may be requested from the corresponding author.

## Abbreviations

CCK-8: Cell Counting Kit-8
ceRNA: Competing endogenous RNA
IGF2BP2: Insulin-like growth factor 2 mRNA-binding protein 2
IHC: Immunohistochemistry
lncRNA: Long noncoding RNA
miRNA: MicroRNA
OS: Overall survival
qRT-PCR: Quantitative reverse transcription polymerase chain reaction
RIP: RNA immunoprecipitation
