# Supplementary material for: Long noncoding RNA LIPH-4 promotes esophageal squamous cell carcinoma progression by regulating the miR-216b/IGF2BP2 axis
Source: Biomark Res. 2022 Aug 16;10:60. doi: 10.1186/s40364-022-00408-x (PMC9380392; doi:10.1186/s40364-022-00408-x)
Supplement: Supplementary file 1 — Additional file 1: Supplementary Table 1. Primer sequences for qRT-PCR. Supplementary Table 2. Sequences of siRNA. [file 40364_2022_408_MOESM1_ESM.doc]

**Supplementary Table 1.** Primer sequences for qRT-PCR.

| Gene | Forward sequence (5'-3') | Reverse sequence (5'-3') |
| --- | --- | --- |
| LIPH-4 | GCCTGTGCCAGTGCTGAGATAG | GAAAACCAGCGGAGAAGAAAGC |
| IGF2BP2 | AGCTAAGCGGGCATCAGTTTG | CCGCAGCGGGAAATCAATCT |
| GAPDH | ACAGCCTCAAGATCATCAGC | GGTCATGAGTCCTTCCACGAT |

**Supplementary Table 2.** Sequences of siRNA

| Name | Sequence |
| --- | --- |
| si-NC | TTCTCCGAACGTGTCACGT |
| si-LIPH-4#1 | ATCAGGAAACCAACTAGTAAA |
| si-LIPH-4#2 | CAGCTAATGAGGAAGTGCCTT |
| si-IGF2BP2 | CGCAAGATCAGGGAAATTG |
